# Carbon­yl[tris­(3,5-diphenyl­pyrazol-1-yl-κ*N* ^2^)methane]copper(I) hexa­fluorido­phosphate–dichloro­methane–diethyl ether (4/3/1)

**DOI:** 10.1107/S1600536809040781

**Published:** 2009-10-13

**Authors:** Katie E. Miller, Lauren M. Schopp, Kelly N. Nesseth, Curtis Moore, Arnold L. Rheingold, Christopher J. A. Daley

**Affiliations:** a Department of Chemistry and Biochemistry, University of San Diego, 5998 Alcalá Park, San Diego, CA 92110, USA; b Department of Chemistry and Biochemistry, University of California, San Diego, 9500 Gilman Drive, La Jolla, CA 92093, USA

## Abstract

In the title compound, [Cu(C_46_H_34_N_6_)(CO)]PF_6_·0.75CH_2_Cl_2_·0.25C_4_H_10_O, the Cu^I^ atom is coordinated by three N atoms from the tridentate chelating tris­(3,5-diphenyl­pyrazol-1-yl)methane ligand (average Cu—N distance = 2.055 Å) and the C atom from a carbon monoxide ligand in a distorted tetra­hedral coordination geometry. The average N—Cu—N angle between adjacent pyrazole-ring-coordinated N atoms is 88.6°, while the average N—Cu—C angle between the pyrazole-bound N atom and the C atom of carbon monoxide is 126.3°. One of the 3-phenyl rings of the tris­(pyrazol­yl)methane ligand is disordered over two sites each with an occupancy factor of 0.50. The structure also exhibits disorder of the monosolvate that has been modeled with 0.75 CH_2_Cl_2_ and 0.25 Et_2_O occupancy.

## Related literature

For related copper complexes with coordinated tris­(pyrazol­yl)­methane ligands, see: Kujime *et al.* (2007 ▶); Fujisawa *et al.* (2006 ▶).
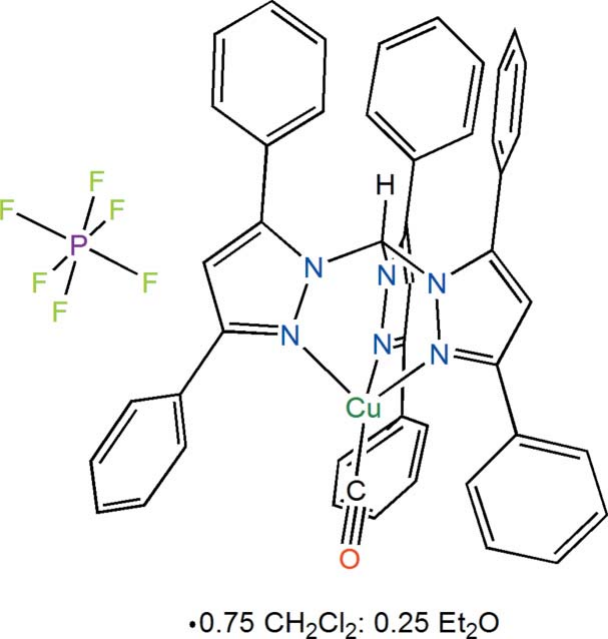


## Experimental

### 

#### Crystal data

[Cu(C_46_H_34_N_6_)(CO)]PF_6_·0.75CH_2_Cl_2_·0.25C_4_H_10_O
*M* *_r_* = 989.54
Monoclinic, 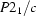

*a* = 19.891 (3) Å
*b* = 13.772 (2) Å
*c* = 16.091 (3) Å
β = 93.847 (2)°
*V* = 4398.0 (13) Å^3^
*Z* = 4
Mo *K*α radiation
μ = 0.70 mm^−1^
*T* = 150 K
0.26 × 0.16 × 0.11 mm

#### Data collection

Bruker APEXII CCD diffractometer
Absorption correction: multi-scan (*SADABS*; Bruker, 2007 ▶) *T*_min_ = 0.840, *T*_max_ = 0.927
57538 measured reflections
10230 independent reflections
7692 reflections with *I* > 2σ(*I*)
*R*_int_ = 0.036

#### Refinement

*R*[*F* ^2^ > 2σ(*F* ^2^)] = 0.043
*wR*(*F* ^2^) = 0.116
*S* = 1.04
10230 reflections
655 parameters
6 restraints
H-atom parameters constrained
Δρ_max_ = 1.02 e Å^−3^
Δρ_min_ = −0.85 e Å^−3^

### 

Data collection: *APEX2* (Bruker, 2007 ▶); cell refinement: *SAINT* (Bruker, 2007 ▶); data reduction: *SAINT*; program(s) used to solve structure: *SHELXS97* (Sheldrick, 2008 ▶); program(s) used to refine structure: *SHELXL97* (Sheldrick, 2008 ▶); molecular graphics: *ORTEP-3* (Farrugia, 1997 ▶); software used to prepare material for publication: *publCIF* (Westrip, 2009 ▶).

## Supplementary Material

Crystal structure: contains datablocks I, global. DOI: 10.1107/S1600536809040781/wm2257sup1.cif

Structure factors: contains datablocks I. DOI: 10.1107/S1600536809040781/wm2257Isup2.hkl

Additional supplementary materials:  crystallographic information; 3D view; checkCIF report

## Figures and Tables

**Table 1:** Selected bond lengths (Å)

C1—Cu1	1.796 (3)
Cu1—N6	2.0453 (18)
Cu1—N2	2.0588 (18)
Cu1—N4	2.0617 (19)

**Fig. 1.**
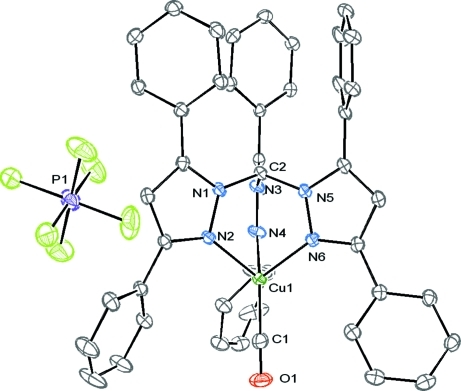
Perspective view of complex [1]PF6. Displacement ellipsoids are drawn at the 30% probability level. H-atoms and disordered solvate molecules are omitted for clarity. Only one of the two conformations (50:50) of the disordered main molecule is depicted for clarity. The disorder is in the 3-phenyl ring of one of the the 3,5-diphenylpyrazole arms of the tris(pyrazolyl)methane ligand.

## supplementary crystallographic information

### Comment

In the course of studying the chemistry of tris(pyrazolyl)methane copper(I) complexes, we reacted several substituted tris(pyrazolyl)methane (Tpm) ligands with various copper(I) salts to form the corresponding [Cu(Tpm)]^+^ complexes based on literature references. Once prepared, we tested the complexes for activity as catalysts, and we examined their reactivity with CO. The latter has been performed on several [Cu(Tpm)]^+^ complexes including η^3^-tris(3,5-diphenylpyrazolyl)methane copper(I) perchlorate and has yielded the expected monocarbonyl adducts [Cu(Tpm)(CO)]^+^ (Kujime *et al.*, 2007). While the synthesis of [Cu(^3,5-Ph^Tpm)]ClO_4_ ([1]ClO_4_) has been reported, its crystal structure has not been determined. As such, we prepared the hexafluoridophospate salt analogue (avoiding the potentially dangerous perchlorate salt) and report its crystal structure.

The crystal structure of [1]PF_6_, is shown in Figure 1. The Cu^I^ atom is four-coordinate, bound by 3 N atoms from the tris(pyrazolyl)methane ligand and the C atom of carbon monoxide in a distorted tetrahedral coordination geometry. The average Cu—N bond distances (2.055 Å), Cu—C bond distance (1.796 (3) Å), and C—O bond distance (1.126 (3) Å) are within normal ranges as are the average N—Cu—N angles from adjacent pyrazolyl arms (88.6 Å), average N—Cu—C angles from bound pyrazolyl N atom to carbon monoxide C atom (126.3 Å), and the Cu—C—O bond angle (179.59 (3) Å) (Fujisawa *et al.*, 2006).

### Experimental

Ligand HC(3,5-Ph_2_py)_3_ (0.100 g, 0.149 mmol) was added to tetrakis(acetonitrile)copper(I) hexafluoridophosphate (0.0557 g, 0.149 mmol) in methylene chloride (10 ml) under N_2_ atmosphere in a 50 ml Schlenk flask. The mixture was stirred for 2 h then reacted with CO by bubbling CO_(_*_g_*_)_, prepared from the reaction of concentrated sulfuric acid and formic acid, through the solution for 10 min. The flask was left under CO atmosphere for 2 d. The flask was opened to N_2_ atmosphere again and hexane (18 ml) was added to precipitate the product. The product was isolated by inverse filtration and dried under a stream of N_2_ (112 mg, 0.123 mmol, 82.8%). FTIR analysis showed the expected strong νCO peak at 2098 cm^-1.^ Single crystals were obtained by vapor diffusion of diethyl ether into a concentrated CH_2_Cl_2_ solution of [1]PF_6_ at room temperature over several days.

### Refinement

All hydrogen atoms were included at idealized positions and treated as riding to their parent atoms. The solvent in the lattice was modeled with a 75/25 disorder of dichloromethan/diethyl ether. The rotational disorder in the phenyl ring was modeled as a 50/50 disorder.

### Crystal data

**Table:**

[Cu(C_46_H_34_N_6_)(CO)]PF_6_·0.75CH_2_Cl_2_·0.25C_4_H_10_O	*F*(000) = 2024
*M**_r_* = 989.54	*D*_x_ = 1.494 Mg m^−^^3^
Monoclinic, *P*2_1_/*c*	Mo *K*α radiation, λ = 0.71073 Å
Hall symbol: -P 2ybc	Cell parameters from 9980 reflections
*a* = 19.891 (3) Å	θ = 2.2–24.9°
*b* = 13.772 (2) Å	µ = 0.70 mm^−^^1^
*c* = 16.091 (3) Å	*T* = 150 K
β = 93.847 (2)°	Needle, colourless
*V* = 4398.0 (13) Å^3^	0.26 × 0.16 × 0.11 mm
*Z* = 4	

### Data collection

**Table:**

Bruker APEXII CCD diffractometer	10230 independent reflections
Radiation source: fine-focus sealed tube	7692 reflections with *I* > 2σ(*I*)
graphite	*R*_int_ = 0.036
φ and ω scans	θ_max_ = 28.2°, θ_min_ = 1.8°
Absorption correction: multi-scan (*SADABS*; Bruker, 2007)	*h* = −25→25
*T*_min_ = 0.840, *T*_max_ = 0.927	*k* = −18→17
57538 measured reflections	*l* = −21→21

### Refinement

**Table:**

Refinement on *F*^2^	Primary atom site location: structure-invariant direct methods
Least-squares matrix: full	Secondary atom site location: difference Fourier map
*R*[*F*^2^ > 2σ(*F*^2^)] = 0.043	Hydrogen site location: inferred from neighbouring sites
*wR*(*F*^2^) = 0.116	H-atom parameters constrained
*S* = 1.04	*w* = 1/[σ^2^(*F*_o_^2^) + (0.0529*P*)^2^ + 3.6625*P*] where *P* = (*F*_o_^2^ + 2*F*_c_^2^)/3
10230 reflections	(Δ/σ)_max_ = 0.001
655 parameters	Δρ_max_ = 1.02 e Å^−^^3^
6 restraints	Δρ_min_ = −0.84 e Å^−^^3^

### Special details

**Table:**

Geometry. All s.u.'s (except the s.u. in the dihedral angle between two l.s. planes) are estimated using the full covariance matrix. The cell s.u.'s are taken into account individually in the estimation of s.u.'s in distances, angles and torsion angles; correlations between s.u.'s in cell parameters are only used when they are defined by crystal symmetry. An approximate (isotropic) treatment of cell s.u.'s is used for estimating s.u.'s involving l.s. planes.
Refinement. Refinement of *F*^2^ against ALL reflections. The weighted *R*-factor *wR* and goodness of fit *S* are based on *F*^2^, conventional *R*-factors *R* are based on *F*, with *F* set to zero for negative *F*^2^. The threshold expression of *F*^2^ > 2σ(*F*^2^) is used only for calculating *R*-factors(gt) *etc*. and is not relevant to the choice of reflections for refinement. *R*-factors based on *F*^2^ are statistically about twice as large as those based on *F*, and *R*- factors based on ALL data will be even larger.

### Fractional atomic coordinates and isotropic or equivalent isotropic displacement parameters (Å^2^)

**Table:**

	*x*	*y*	*z*	*U*_iso_*/*U*_eq_	Occ. (<1)
C1	0.42242 (13)	0.21405 (19)	0.37258 (16)	0.0355 (6)	
C2	0.21856 (10)	0.23550 (15)	0.51521 (13)	0.0214 (4)	
H2	0.1759	0.2386	0.5447	0.026*	
C3	0.19736 (10)	0.41694 (15)	0.49359 (13)	0.0222 (4)	
C4	0.23539 (11)	0.48860 (16)	0.45989 (13)	0.0247 (4)	
H4	0.2249	0.5559	0.4566	0.030*	
C5	0.29246 (10)	0.44312 (15)	0.43141 (13)	0.0227 (4)	
C6	0.13438 (10)	0.42759 (15)	0.53555 (13)	0.0231 (4)	
C7	0.07862 (11)	0.36688 (16)	0.51896 (14)	0.0255 (5)	
H7	0.0805	0.3164	0.4789	0.031*	
C8	0.02067 (11)	0.38043 (17)	0.56094 (15)	0.0308 (5)	
H8	−0.0170	0.3389	0.5499	0.037*	
C9	0.01761 (12)	0.45438 (17)	0.61897 (16)	0.0330 (5)	
H9	−0.0219	0.4626	0.6483	0.040*	
C10	0.07186 (12)	0.51634 (17)	0.63438 (16)	0.0321 (5)	
H10	0.0693	0.5677	0.6734	0.039*	
C11	0.13009 (11)	0.50329 (16)	0.59263 (14)	0.0276 (5)	
H11	0.1672	0.5461	0.6030	0.033*	
C12	0.34983 (11)	0.48851 (16)	0.39286 (14)	0.0260 (5)	
C13	0.33862 (13)	0.56580 (18)	0.33750 (16)	0.0346 (5)	
H13	0.2942	0.5893	0.3248	0.042*	
C14	0.39264 (15)	0.6081 (2)	0.3011 (2)	0.0538 (8)	
H14	0.3850	0.6599	0.2626	0.065*	
C15	0.45756 (16)	0.5755 (3)	0.3205 (2)	0.0649 (10)	
H15	0.4943	0.6046	0.2951	0.078*	
C16	0.46896 (14)	0.5008 (2)	0.3766 (2)	0.0508 (8)	
H16	0.5137	0.4792	0.3905	0.061*	
C17	0.41550 (12)	0.45705 (18)	0.41269 (15)	0.0313 (5)	
H17	0.4236	0.4054	0.4512	0.038*	
C18	0.26913 (11)	0.17693 (15)	0.65675 (13)	0.0234 (4)	
C19	0.33265 (11)	0.14563 (16)	0.68269 (14)	0.0267 (5)	
H19	0.3467	0.1208	0.7362	0.032*	
C20	0.37271 (11)	0.15735 (19)	0.61518 (15)	0.0318 (5)	
C21	0.20807 (10)	0.18089 (16)	0.70288 (13)	0.0225 (4)	
C22	0.16647 (11)	0.26249 (16)	0.70177 (13)	0.0247 (4)	
H22	0.1780	0.3188	0.6717	0.030*	
C23	0.10811 (12)	0.26144 (17)	0.74472 (14)	0.0279 (5)	
H23	0.0792	0.3164	0.7427	0.034*	
C24	0.09191 (12)	0.18034 (18)	0.79061 (14)	0.0299 (5)	
H24	0.0519	0.1797	0.8197	0.036*	
C25	0.13437 (11)	0.10012 (17)	0.79392 (14)	0.0282 (5)	
H25	0.1239	0.0452	0.8264	0.034*	
C26	0.19178 (11)	0.09996 (16)	0.75005 (13)	0.0256 (5)	
H26	0.2203	0.0446	0.7519	0.031*	
C27	0.44506 (13)	0.1360 (3)	0.61176 (18)	0.0606 (10)	
C28	0.4780 (3)	0.0837 (5)	0.6667 (4)	0.0355 (13)	0.50
H28	0.4565	0.0434	0.7049	0.043*	0.50
C29	0.55288 (17)	0.0914 (4)	0.6656 (2)	0.0758 (13)	
H29	0.5782	0.0605	0.7103	0.091*	0.50
H29B	0.5782	0.0798	0.7153	0.091*	0.50
C30	0.5843 (3)	0.1308 (5)	0.6163 (4)	0.0441 (13)	0.50
H30	0.6299	0.1137	0.6098	0.053*	0.50
C31	0.5529 (2)	0.2027 (4)	0.5681 (4)	0.0408 (12)	0.50
H31	0.5783	0.2428	0.5337	0.049*	0.50
C32	0.4840 (2)	0.2157 (4)	0.5706 (3)	0.0335 (11)	0.50
H32	0.4621	0.2719	0.5476	0.040*	0.50
C33	0.15501 (11)	0.10446 (16)	0.43094 (13)	0.0239 (4)	
C34	0.17172 (11)	0.04769 (16)	0.36559 (14)	0.0255 (5)	
H34	0.1447	−0.0014	0.3388	0.031*	
C35	0.23664 (10)	0.07607 (15)	0.34583 (13)	0.0231 (4)	
C36	0.09176 (11)	0.11060 (16)	0.47349 (14)	0.0273 (5)	
C37	0.08969 (14)	0.0902 (2)	0.55808 (15)	0.0402 (6)	
H37	0.1294	0.0709	0.5898	0.048*	
C38	0.02932 (17)	0.0984 (2)	0.59565 (18)	0.0534 (9)	
H38	0.0278	0.0846	0.6533	0.064*	
C39	−0.02860 (16)	0.1264 (2)	0.5501 (2)	0.0519 (8)	
H39	−0.0695	0.1334	0.5768	0.062*	
C40	−0.02732 (13)	0.14420 (19)	0.46590 (19)	0.0431 (7)	
H40	−0.0674	0.1625	0.4344	0.052*	
C41	0.03278 (11)	0.13529 (17)	0.42740 (16)	0.0307 (5)	
H41	0.0335	0.1462	0.3692	0.037*	
C42	0.27480 (10)	0.03898 (16)	0.27714 (13)	0.0235 (4)	
C43	0.26789 (11)	−0.05816 (17)	0.25368 (14)	0.0277 (5)	
H43	0.2413	−0.1007	0.2844	0.033*	
C44	0.29983 (12)	−0.09300 (18)	0.18539 (15)	0.0318 (5)	
H44	0.2953	−0.1593	0.1697	0.038*	
C45	0.33828 (12)	−0.0309 (2)	0.14024 (15)	0.0341 (5)	
H45	0.3593	−0.0545	0.0930	0.041*	
C46	0.31462 (11)	0.10025 (17)	0.23170 (14)	0.0273 (5)	
H46	0.3201	0.1664	0.2476	0.033*	
C47	0.34620 (11)	0.06525 (19)	0.16361 (15)	0.0319 (5)	
H47	0.3733	0.1073	0.1330	0.038*	
C28B	0.4911 (3)	0.1379 (5)	0.6694 (4)	0.0382 (13)	0.50
H28B	0.4831	0.1732	0.7185	0.046*	0.50
C30B	0.5773 (3)	0.0603 (5)	0.5966 (4)	0.0418 (13)	0.50
H30B	0.6243	0.0544	0.5906	0.050*	0.50
C31B	0.5304 (3)	0.0371 (4)	0.5340 (4)	0.0484 (14)	0.50
H31B	0.5436	0.0017	0.4871	0.058*	0.50
C32B	0.4634 (3)	0.0646 (4)	0.5380 (3)	0.0378 (12)	0.50
H32B	0.4296	0.0419	0.4982	0.045*	0.50
C1S	0.7723 (3)	0.1515 (4)	0.4724 (4)	0.0513 (12)	0.75
H1S1	0.7971	0.1754	0.4251	0.062*	0.75
H1S2	0.8058	0.1311	0.5173	0.062*	0.75
C2S	0.5816	0.2881	0.4872	0.033 (2)	0.25
H2S1	0.5755	0.3054	0.4281	0.049*	0.25
H2S2	0.5692	0.3435	0.5212	0.049*	0.25
H2S3	0.5527	0.2325	0.4984	0.049*	0.25
C3S	0.6585	0.2600	0.5098	0.032 (2)	0.25
H3S1	0.6882	0.3160	0.4999	0.039*	0.25
H3S2	0.6651	0.2411	0.5692	0.039*	0.25
C4S	0.7439	0.1566	0.4766	0.047 (4)	0.25
H4S1	0.7560	0.1501	0.5370	0.056*	0.25
H4S2	0.7746	0.2039	0.4524	0.056*	0.25
C5S	0.7455	0.0600	0.4331	0.057 (6)	0.25
H5S1	0.7248	0.0665	0.3763	0.086*	0.25
H5S2	0.7204	0.0121	0.4637	0.086*	0.25
H5S3	0.7923	0.0386	0.4308	0.086*	0.25
Cl1S	0.72306 (8)	0.05154 (8)	0.44009 (9)	0.0617 (3)	0.75
Cl2S	0.72256 (7)	0.24608 (9)	0.50909 (8)	0.0716 (4)	0.75
Cu1	0.346041 (13)	0.224475 (19)	0.425401 (16)	0.02380 (8)	
F1	0.13767 (9)	0.81255 (12)	0.31085 (11)	0.0542 (4)	
F2	0.05916 (8)	0.71373 (14)	0.24983 (13)	0.0632 (5)	
F3	0.14332 (9)	0.77162 (11)	0.17670 (11)	0.0508 (4)	
F4	0.13903 (12)	0.61327 (12)	0.20844 (14)	0.0730 (6)	
F5	0.21861 (8)	0.71132 (14)	0.27099 (14)	0.0688 (6)	
F6	0.13469 (10)	0.65282 (14)	0.34508 (12)	0.0659 (5)	
N1	0.23144 (8)	0.33174 (13)	0.48265 (11)	0.0216 (4)	
N2	0.29039 (9)	0.34751 (13)	0.44469 (11)	0.0223 (4)	
N3	0.27266 (9)	0.20641 (13)	0.57560 (11)	0.0235 (4)	
N4	0.33619 (9)	0.19362 (15)	0.54937 (12)	0.0299 (4)	
N5	0.20926 (8)	0.16426 (13)	0.44889 (11)	0.0222 (4)	
N6	0.25974 (8)	0.14726 (13)	0.39678 (11)	0.0224 (4)	
O1	0.47013 (10)	0.20716 (18)	0.33911 (14)	0.0588 (6)	
O1S	0.6733	0.1850	0.4606	0.0355 (15)	0.25
P1	0.13840 (3)	0.71233 (5)	0.26125 (5)	0.03739 (17)	

### Atomic displacement parameters (Å^2^)

**Table:**

	*U*^11^	*U*^22^	*U*^33^	*U*^12^	*U*^13^	*U*^23^
C1	0.0317 (13)	0.0377 (14)	0.0369 (14)	−0.0027 (10)	0.0024 (11)	−0.0076 (11)
C2	0.0194 (10)	0.0213 (10)	0.0234 (11)	−0.0032 (8)	−0.0002 (8)	0.0009 (8)
C3	0.0219 (10)	0.0213 (10)	0.0227 (11)	−0.0008 (8)	−0.0034 (8)	−0.0013 (8)
C4	0.0247 (11)	0.0211 (10)	0.0276 (11)	−0.0023 (8)	−0.0024 (9)	0.0001 (9)
C5	0.0244 (10)	0.0229 (11)	0.0204 (10)	−0.0041 (8)	−0.0010 (8)	−0.0001 (8)
C6	0.0212 (10)	0.0216 (10)	0.0263 (11)	0.0022 (8)	0.0008 (8)	0.0032 (9)
C7	0.0235 (10)	0.0224 (11)	0.0303 (12)	−0.0003 (8)	−0.0002 (9)	0.0012 (9)
C8	0.0227 (11)	0.0270 (12)	0.0424 (14)	−0.0024 (9)	0.0000 (10)	0.0051 (10)
C9	0.0269 (11)	0.0287 (12)	0.0444 (14)	0.0040 (9)	0.0093 (10)	0.0041 (11)
C10	0.0313 (12)	0.0270 (12)	0.0385 (13)	0.0045 (10)	0.0050 (10)	−0.0040 (10)
C11	0.0234 (11)	0.0227 (11)	0.0364 (13)	−0.0018 (9)	0.0005 (9)	−0.0012 (9)
C12	0.0289 (11)	0.0233 (11)	0.0258 (11)	−0.0055 (9)	0.0028 (9)	−0.0013 (9)
C13	0.0352 (13)	0.0306 (13)	0.0378 (14)	−0.0043 (10)	0.0005 (10)	0.0070 (11)
C14	0.0532 (18)	0.0515 (18)	0.0575 (19)	−0.0080 (14)	0.0101 (14)	0.0292 (15)
C15	0.0399 (16)	0.074 (2)	0.082 (2)	−0.0115 (15)	0.0181 (16)	0.039 (2)
C16	0.0281 (13)	0.0574 (18)	0.068 (2)	−0.0051 (12)	0.0105 (13)	0.0219 (16)
C17	0.0294 (12)	0.0315 (12)	0.0331 (13)	−0.0055 (10)	0.0027 (10)	0.0049 (10)
C18	0.0278 (11)	0.0199 (10)	0.0224 (11)	−0.0025 (8)	0.0005 (8)	−0.0011 (8)
C19	0.0277 (11)	0.0270 (11)	0.0250 (11)	−0.0021 (9)	−0.0019 (9)	0.0025 (9)
C20	0.0222 (11)	0.0410 (14)	0.0317 (12)	−0.0030 (10)	−0.0019 (9)	0.0079 (11)
C21	0.0245 (10)	0.0232 (11)	0.0196 (10)	−0.0020 (8)	−0.0011 (8)	−0.0025 (8)
C22	0.0303 (11)	0.0222 (11)	0.0214 (11)	−0.0018 (9)	−0.0004 (9)	−0.0002 (8)
C23	0.0308 (12)	0.0285 (12)	0.0244 (11)	0.0044 (9)	0.0008 (9)	−0.0037 (9)
C24	0.0304 (12)	0.0366 (13)	0.0228 (11)	−0.0005 (10)	0.0040 (9)	−0.0019 (10)
C25	0.0317 (12)	0.0311 (12)	0.0220 (11)	−0.0041 (10)	0.0027 (9)	0.0031 (9)
C26	0.0283 (11)	0.0242 (11)	0.0238 (11)	0.0000 (9)	−0.0020 (9)	0.0004 (9)
C27	0.0233 (13)	0.118 (3)	0.0404 (16)	0.0085 (15)	0.0027 (11)	0.0355 (18)
C28	0.035 (3)	0.038 (3)	0.033 (3)	0.005 (3)	−0.003 (2)	0.002 (3)
C29	0.0461 (18)	0.148 (4)	0.0318 (16)	0.048 (2)	−0.0068 (14)	0.003 (2)
C30	0.028 (3)	0.063 (4)	0.041 (3)	0.008 (3)	−0.003 (2)	−0.002 (3)
C31	0.025 (2)	0.051 (3)	0.046 (3)	0.000 (2)	−0.001 (2)	0.009 (3)
C32	0.022 (2)	0.037 (3)	0.041 (3)	0.0022 (19)	−0.0028 (19)	0.009 (2)
C33	0.0236 (10)	0.0226 (10)	0.0250 (11)	−0.0050 (8)	−0.0014 (8)	0.0020 (9)
C34	0.0271 (11)	0.0232 (11)	0.0258 (11)	−0.0047 (9)	−0.0008 (9)	−0.0006 (9)
C35	0.0236 (10)	0.0205 (10)	0.0247 (11)	0.0007 (8)	−0.0019 (8)	0.0013 (8)
C36	0.0280 (11)	0.0248 (11)	0.0295 (12)	−0.0110 (9)	0.0055 (9)	−0.0047 (9)
C37	0.0488 (15)	0.0441 (15)	0.0280 (13)	−0.0272 (12)	0.0042 (11)	−0.0031 (11)
C38	0.068 (2)	0.0612 (19)	0.0336 (15)	−0.0426 (17)	0.0218 (14)	−0.0164 (14)
C39	0.0539 (18)	0.0451 (17)	0.061 (2)	−0.0272 (14)	0.0381 (16)	−0.0226 (15)
C40	0.0325 (13)	0.0354 (14)	0.0636 (19)	−0.0077 (11)	0.0190 (12)	−0.0048 (13)
C41	0.0294 (12)	0.0284 (12)	0.0353 (13)	−0.0065 (9)	0.0090 (10)	0.0005 (10)
C42	0.0212 (10)	0.0260 (11)	0.0225 (11)	0.0008 (8)	−0.0038 (8)	0.0007 (9)
C43	0.0243 (11)	0.0288 (12)	0.0292 (12)	−0.0030 (9)	−0.0036 (9)	−0.0029 (9)
C44	0.0287 (12)	0.0340 (13)	0.0320 (13)	0.0007 (10)	−0.0033 (9)	−0.0095 (10)
C45	0.0259 (11)	0.0501 (15)	0.0259 (12)	0.0073 (11)	−0.0008 (9)	−0.0047 (11)
C46	0.0231 (11)	0.0260 (11)	0.0324 (12)	0.0023 (9)	−0.0020 (9)	0.0044 (9)
C47	0.0246 (11)	0.0425 (14)	0.0284 (12)	0.0021 (10)	0.0005 (9)	0.0070 (11)
C28B	0.032 (3)	0.055 (4)	0.027 (3)	0.006 (3)	0.001 (2)	−0.006 (3)
C30B	0.025 (3)	0.052 (4)	0.047 (3)	0.013 (2)	−0.003 (2)	0.002 (3)
C31B	0.041 (3)	0.053 (4)	0.051 (3)	0.016 (3)	0.002 (3)	−0.018 (3)
C32B	0.034 (3)	0.036 (3)	0.043 (3)	0.010 (2)	−0.004 (2)	−0.013 (2)
C1S	0.044 (3)	0.043 (3)	0.067 (3)	−0.0053 (19)	0.002 (2)	0.005 (2)
C2S	0.033 (5)	0.027 (5)	0.038 (5)	−0.004 (4)	0.002 (4)	0.007 (4)
C3S	0.034 (5)	0.028 (5)	0.034 (5)	−0.013 (4)	−0.005 (4)	0.007 (4)
C4S	0.070 (12)	0.039 (7)	0.032 (6)	0.017 (7)	0.007 (7)	0.003 (5)
C5S	0.067 (7)	0.055 (7)	0.048 (6)	0.020 (4)	−0.009 (4)	−0.002 (4)
Cl1S	0.0581 (7)	0.0341 (6)	0.0923 (10)	−0.0055 (5)	0.0016 (6)	−0.0124 (6)
Cl2S	0.0942 (9)	0.0459 (6)	0.0776 (8)	−0.0050 (6)	0.0261 (7)	−0.0174 (6)
Cu1	0.02001 (13)	0.02522 (15)	0.02630 (15)	−0.00240 (10)	0.00253 (10)	−0.00122 (11)
F1	0.0586 (10)	0.0414 (9)	0.0659 (11)	−0.0103 (8)	0.0290 (9)	−0.0078 (8)
F2	0.0290 (8)	0.0748 (13)	0.0853 (14)	−0.0085 (8)	−0.0008 (8)	0.0205 (11)
F3	0.0631 (11)	0.0338 (9)	0.0574 (10)	0.0033 (7)	0.0177 (8)	0.0088 (7)
F4	0.1047 (16)	0.0279 (9)	0.0863 (15)	0.0005 (9)	0.0050 (12)	0.0018 (9)
F5	0.0298 (9)	0.0645 (12)	0.1123 (17)	0.0112 (8)	0.0067 (9)	0.0202 (11)
F6	0.0656 (12)	0.0604 (12)	0.0696 (12)	−0.0195 (9)	−0.0107 (9)	0.0325 (10)
N1	0.0198 (8)	0.0204 (9)	0.0247 (9)	−0.0040 (7)	0.0020 (7)	0.0007 (7)
N2	0.0210 (9)	0.0237 (9)	0.0225 (9)	−0.0047 (7)	0.0034 (7)	−0.0004 (7)
N3	0.0180 (8)	0.0275 (10)	0.0247 (9)	−0.0022 (7)	−0.0005 (7)	0.0028 (7)
N4	0.0176 (9)	0.0416 (11)	0.0305 (10)	−0.0011 (8)	0.0010 (7)	0.0069 (9)
N5	0.0208 (8)	0.0210 (9)	0.0248 (9)	−0.0028 (7)	0.0017 (7)	−0.0004 (7)
N6	0.0210 (9)	0.0211 (9)	0.0251 (9)	0.0010 (7)	0.0020 (7)	−0.0004 (7)
O1	0.0364 (11)	0.0804 (16)	0.0622 (14)	−0.0048 (10)	0.0235 (10)	−0.0186 (12)
O1S	0.031 (3)	0.039 (4)	0.036 (4)	0.006 (3)	0.003 (3)	0.002 (3)
P1	0.0277 (3)	0.0267 (3)	0.0579 (4)	0.0015 (2)	0.0043 (3)	0.0107 (3)

### Geometric parameters (Å, °)

**Table:**

C1—O1	1.126 (3)	C31—H31	0.9500
C1—Cu1	1.796 (3)	C32—H32	0.9500
C2—N5	1.452 (3)	C33—C34	1.369 (3)
C2—N1	1.454 (3)	C33—N5	1.373 (3)
C2—N3	1.457 (3)	C33—C36	1.475 (3)
C2—H2	1.0000	C34—C35	1.406 (3)
C3—N1	1.372 (3)	C34—H34	0.9500
C3—C4	1.377 (3)	C35—N6	1.339 (3)
C3—C6	1.470 (3)	C35—C42	1.474 (3)
C4—C5	1.400 (3)	C36—C41	1.388 (3)
C4—H4	0.9500	C36—C37	1.393 (3)
C5—N2	1.335 (3)	C37—C38	1.385 (4)
C5—C12	1.474 (3)	C37—H37	0.9500
C6—C11	1.396 (3)	C38—C39	1.378 (5)
C6—C7	1.400 (3)	C38—H38	0.9500
C7—C8	1.387 (3)	C39—C40	1.379 (4)
C7—H7	0.9500	C39—H39	0.9500
C8—C9	1.386 (3)	C40—C41	1.388 (3)
C8—H8	0.9500	C40—H40	0.9500
C9—C10	1.385 (3)	C41—H41	0.9500
C9—H9	0.9500	C42—C43	1.394 (3)
C10—C11	1.389 (3)	C42—C46	1.397 (3)
C10—H10	0.9500	C43—C44	1.391 (3)
C11—H11	0.9500	C43—H43	0.9500
C12—C17	1.393 (3)	C44—C45	1.385 (4)
C12—C13	1.396 (3)	C44—H44	0.9500
C13—C14	1.386 (4)	C45—C47	1.383 (4)
C13—H13	0.9500	C45—H45	0.9500
C14—C15	1.383 (4)	C46—C47	1.385 (3)
C14—H14	0.9500	C46—H46	0.9500
C15—C16	1.376 (4)	C47—H47	0.9500
C15—H15	0.9500	C28B—H28B	0.9500
C16—C17	1.384 (3)	C30B—C31B	1.364 (8)
C16—H16	0.9500	C30B—H30B	0.9500
C17—H17	0.9500	C31B—C32B	1.390 (7)
C18—C19	1.373 (3)	C31B—H31B	0.9500
C18—N3	1.374 (3)	C32B—H32B	0.9500
C18—C21	1.466 (3)	C1S—Cl1S	1.748 (5)
C19—C20	1.399 (3)	C1S—Cl2S	1.762 (5)
C19—H19	0.9500	C1S—H1S1	0.9900
C20—N4	1.340 (3)	C1S—H1S2	0.9900
C20—C27	1.473 (3)	C2S—C3S	1.5972 (2)
C21—C22	1.395 (3)	C2S—H2S1	0.9800
C21—C26	1.399 (3)	C2S—H2S2	0.9800
C22—C23	1.391 (3)	C2S—H2S3	0.9800
C22—H22	0.9500	C3S—O1S	1.34576 (16)
C23—C24	1.389 (3)	C3S—H3S1	0.9900
C23—H23	0.9500	C3S—H3S2	0.9900
C24—C25	1.389 (3)	C4S—O1S	1.4641 (2)
C24—H24	0.9500	C4S—C5S	1.5050 (2)
C25—C26	1.382 (3)	C4S—H4S1	0.9900
C25—H25	0.9500	C4S—H4S2	0.9900
C26—H26	0.9500	C5S—H5S1	0.9800
C27—C28B	1.259 (6)	C5S—H5S2	0.9800
C27—C28	1.286 (6)	C5S—H5S3	0.9800
C27—C32	1.521 (6)	Cu1—N6	2.0453 (18)
C27—C32B	1.602 (6)	Cu1—N2	2.0588 (18)
C28—C29	1.494 (7)	Cu1—N4	2.0617 (19)
C28—H28	0.9500	F1—P1	1.5949 (18)
C29—C30	1.175 (7)	F2—P1	1.5747 (17)
C29—C30B	1.313 (7)	F3—P1	1.5953 (18)
C29—C28B	1.391 (6)	F4—P1	1.608 (2)
C29—H29	0.9500	F5—P1	1.5928 (18)
C29—H29B	0.9300	F6—P1	1.5842 (18)
C30—C31	1.381 (8)	N1—N2	1.375 (2)
C30—H30	0.9500	N3—N4	1.370 (2)
C31—C32	1.385 (7)	N5—N6	1.371 (2)
			
O1—C1—Cu1	179.6 (3)	C34—C35—C42	126.90 (19)
N5—C2—N1	111.58 (17)	C41—C36—C37	119.5 (2)
N5—C2—N3	110.88 (17)	C41—C36—C33	118.9 (2)
N1—C2—N3	110.53 (16)	C37—C36—C33	121.6 (2)
N5—C2—H2	107.9	C38—C37—C36	119.6 (3)
N1—C2—H2	107.9	C38—C37—H37	120.2
N3—C2—H2	107.9	C36—C37—H37	120.2
N1—C3—C4	105.85 (18)	C39—C38—C37	120.6 (3)
N1—C3—C6	126.01 (19)	C39—C38—H38	119.7
C4—C3—C6	128.1 (2)	C37—C38—H38	119.7
C3—C4—C5	106.69 (19)	C38—C39—C40	120.1 (3)
C3—C4—H4	126.7	C38—C39—H39	119.9
C5—C4—H4	126.7	C40—C39—H39	119.9
N2—C5—C4	110.76 (19)	C39—C40—C41	119.7 (3)
N2—C5—C12	121.24 (19)	C39—C40—H40	120.1
C4—C5—C12	128.0 (2)	C41—C40—H40	120.1
C11—C6—C7	119.3 (2)	C36—C41—C40	120.4 (2)
C11—C6—C3	117.93 (19)	C36—C41—H41	119.8
C7—C6—C3	122.8 (2)	C40—C41—H41	119.8
C8—C7—C6	120.0 (2)	C43—C42—C46	119.1 (2)
C8—C7—H7	120.0	C43—C42—C35	119.3 (2)
C6—C7—H7	120.0	C46—C42—C35	121.5 (2)
C9—C8—C7	120.2 (2)	C44—C43—C42	120.2 (2)
C9—C8—H8	119.9	C44—C43—H43	119.9
C7—C8—H8	119.9	C42—C43—H43	119.9
C10—C9—C8	120.3 (2)	C45—C44—C43	119.9 (2)
C10—C9—H9	119.9	C45—C44—H44	120.0
C8—C9—H9	119.9	C43—C44—H44	120.0
C9—C10—C11	119.9 (2)	C47—C45—C44	120.4 (2)
C9—C10—H10	120.0	C47—C45—H45	119.8
C11—C10—H10	120.0	C44—C45—H45	119.8
C10—C11—C6	120.3 (2)	C47—C46—C42	120.5 (2)
C10—C11—H11	119.8	C47—C46—H46	119.8
C6—C11—H11	119.8	C42—C46—H46	119.8
C17—C12—C13	119.4 (2)	C45—C47—C46	119.9 (2)
C17—C12—C5	120.8 (2)	C45—C47—H47	120.0
C13—C12—C5	119.8 (2)	C46—C47—H47	120.0
C14—C13—C12	119.6 (2)	C27—C28B—C29	124.0 (5)
C14—C13—H13	120.2	C27—C28B—H28B	118.0
C12—C13—H13	120.2	C29—C28B—H28B	118.0
C15—C14—C13	120.4 (3)	C29—C30B—C31B	115.3 (5)
C15—C14—H14	119.8	C29—C30B—H30B	122.4
C13—C14—H14	119.8	C31B—C30B—H30B	122.4
C16—C15—C14	120.1 (3)	C30B—C31B—C32B	120.8 (5)
C16—C15—H15	120.0	C30B—C31B—H31B	119.6
C14—C15—H15	120.0	C32B—C31B—H31B	119.6
C15—C16—C17	120.2 (3)	C31B—C32B—C27	117.9 (4)
C15—C16—H16	119.9	C31B—C32B—H32B	121.0
C17—C16—H16	119.9	C27—C32B—H32B	121.0
C16—C17—C12	120.2 (2)	Cl1S—C1S—Cl2S	111.4 (3)
C16—C17—H17	119.9	Cl1S—C1S—H1S1	109.3
C12—C17—H17	119.9	Cl2S—C1S—H1S1	109.3
C19—C18—N3	106.08 (19)	Cl1S—C1S—H1S2	109.3
C19—C18—C21	129.4 (2)	Cl2S—C1S—H1S2	109.3
N3—C18—C21	124.55 (19)	H1S1—C1S—H1S2	108.0
C18—C19—C20	106.8 (2)	C3S—C2S—H2S1	109.5
C18—C19—H19	126.6	C3S—C2S—H2S2	109.5
C20—C19—H19	126.6	H2S1—C2S—H2S2	109.5
N4—C20—C19	110.5 (2)	C3S—C2S—H2S3	109.5
N4—C20—C27	121.6 (2)	H2S1—C2S—H2S3	109.5
C19—C20—C27	127.9 (2)	H2S2—C2S—H2S3	109.5
C22—C21—C26	119.4 (2)	O1S—C3S—C2S	107.161 (5)
C22—C21—C18	122.3 (2)	O1S—C3S—H3S1	110.3
C26—C21—C18	118.35 (19)	C2S—C3S—H3S1	110.3
C23—C22—C21	120.0 (2)	O1S—C3S—H3S2	110.3
C23—C22—H22	120.0	C2S—C3S—H3S2	110.3
C21—C22—H22	120.0	H3S1—C3S—H3S2	108.5
C24—C23—C22	120.3 (2)	O1S—C4S—C5S	101.746 (4)
C24—C23—H23	119.9	O1S—C4S—H4S1	111.4
C22—C23—H23	119.9	C5S—C4S—H4S1	111.4
C23—C24—C25	119.8 (2)	O1S—C4S—H4S2	111.4
C23—C24—H24	120.1	C5S—C4S—H4S2	111.4
C25—C24—H24	120.1	H4S1—C4S—H4S2	109.3
C26—C25—C24	120.2 (2)	C4S—C5S—H5S1	109.5
C26—C25—H25	119.9	C4S—C5S—H5S2	109.5
C24—C25—H25	119.9	H5S1—C5S—H5S2	109.5
C25—C26—C21	120.3 (2)	C4S—C5S—H5S3	109.5
C25—C26—H26	119.8	H5S1—C5S—H5S3	109.5
C21—C26—H26	119.8	H5S2—C5S—H5S3	109.5
C28B—C27—C20	129.2 (4)	C1—Cu1—N6	125.15 (9)
C28—C27—C20	122.7 (4)	C1—Cu1—N2	128.14 (10)
C28B—C27—C32	86.5 (4)	N6—Cu1—N2	90.59 (7)
C28—C27—C32	116.9 (4)	C1—Cu1—N4	125.44 (10)
C20—C27—C32	113.6 (3)	N6—Cu1—N4	88.73 (7)
C28B—C27—C32B	111.6 (4)	N2—Cu1—N4	86.38 (8)
C28—C27—C32B	92.0 (4)	C3—N1—N2	111.43 (17)
C20—C27—C32B	114.9 (3)	C3—N1—C2	129.23 (17)
C32—C27—C32B	88.4 (3)	N2—N1—C2	118.68 (17)
C27—C28—C29	114.5 (5)	C5—N2—N1	105.25 (17)
C27—C28—H28	122.8	C5—N2—Cu1	139.91 (15)
C29—C28—H28	122.8	N1—N2—Cu1	114.84 (13)
C30—C29—C28B	109.2 (5)	N4—N3—C18	111.21 (17)
C30B—C29—C28B	124.4 (4)	N4—N3—C2	119.02 (17)
C30—C29—C28	128.1 (4)	C18—N3—C2	129.24 (18)
C30B—C29—C28	114.4 (4)	C20—N4—N3	105.48 (18)
C30—C29—H29	116.0	C20—N4—Cu1	139.45 (16)
C30B—C29—H29	106.7	N3—N4—Cu1	114.89 (13)
C28B—C29—H29	126.7	N6—N5—C33	111.50 (17)
C28—C29—H29	116.0	N6—N5—C2	120.16 (16)
C30—C29—H29B	112.1	C33—N5—C2	128.28 (18)
C30B—C29—H29B	117.5	C35—N6—N5	105.31 (17)
C28B—C29—H29B	118.1	C35—N6—Cu1	140.06 (15)
C28—C29—H29B	117.4	N5—N6—Cu1	114.31 (13)
C29—C30—C31	118.0 (5)	C3S—O1S—C4S	110.237 (4)
C29—C30—H30	121.0	F2—P1—F6	90.13 (10)
C31—C30—H30	121.0	F2—P1—F5	178.92 (12)
C30—C31—C32	119.1 (5)	F6—P1—F5	90.88 (11)
C30—C31—H31	120.4	F2—P1—F1	90.30 (10)
C32—C31—H31	120.4	F6—P1—F1	91.10 (11)
C31—C32—C27	117.0 (4)	F5—P1—F1	90.06 (11)
C31—C32—H32	121.5	F2—P1—F3	90.71 (10)
C27—C32—H32	121.5	F6—P1—F3	179.08 (11)
C34—C33—N5	106.05 (19)	F5—P1—F3	88.27 (11)
C34—C33—C36	130.39 (19)	F1—P1—F3	89.25 (9)
N5—C33—C36	123.44 (19)	F2—P1—F4	89.54 (12)
C33—C34—C35	106.79 (19)	F6—P1—F4	90.78 (11)
C33—C34—H34	126.6	F5—P1—F4	90.07 (12)
C35—C34—H34	126.6	F1—P1—F4	178.12 (11)
N6—C35—C34	110.35 (19)	F3—P1—F4	88.88 (10)
N6—C35—C42	122.68 (19)		
